# Obesity-induced hypoadiponectinaemia: the opposite influences of central and peripheral fat compartments

**DOI:** 10.1093/ije/dyx022

**Published:** 2017-03-27

**Authors:** M C Borges, I O Oliveira, D F Freitas, B L Horta, K K Ong, D P Gigante, A J D Barros

**Affiliations:** dyx022-1 Post-Graduate Program in Epidemiology, Federal University of Pelotas, Pelotas, Brazil,; dyx022-2 Department of Physiology and Pharmacology, Federal University of Pelotas, Pelotas, Brazil and; dyx022-3 Medical Research Council (MRC) Epidemiology Unit, University of Cambridge, Cambridge, UK

**Keywords:** Adiponectin, abdominal fat, subcutaneous fat, Mendelian randomization, body fat distribution, adiposity, adipokines

## Abstract

**Background and Aims:**

The substantial reduction in adiponectin concentration among obese individuals seems to depend on fat distribution and is a marker of metabolic and adipose tissue dysfunction. We aimed to: (i) address whether abdominal fat from different compartments (visceral, deep subcutaneous abdominal and superficial subcutaneous abdominal) and gluteofemoral fat are independently associated with blood adiponectin concentration; and (ii) investigate whether abdominal (proxied by waist circumference) and gluteofemoral fat (proxied by hip circumference) accumulation causally determine blood adiponectin concentration.

**Methods:**

To investigate the independent association of abdominal and gluteofemoral fat with adiponectin concentration, we used multivariable regression and data from 30-year-old adults from the 1982 Pelotas Birth Cohort (*n* = 2,743). To assess the causal role of abdominal and gluteofemoral fat accumulation on adiponectin concentration, we used Mendelian randomization and data from two consortia of genome-wide association studies—the GIANT (*n* > 210 000) and ADIPOGen consortia (*n* = 29 347).

**Results:**

In the multivariable regression analysis, all abdominal fat depots were negatively associated with adiponectin concentration, specially visceral abdominal fat [men: β = −0.24 standard unit of log adiponectin per standard unit increase in abdominal fat; 95% confidence interval (CI) = −0.31, −0.18; *P* = 8*10^−13^; women: β = −0.31; 95% CI = −0.36, −0.25; *P* = 7*10^−27^), whereas gluteofemoral fat was positively associated with adiponectin concentration (men: β = 0.13 standard unit of log adiponectin per standard unit increase in gluteofemoral fat; 95% CI = 0.03, 0.22; *P* = 0.008; women: β = 0.24; 95% CI = 0.17, 0.31; *P* = 7*10^−11^). In the Mendelian randomization analysis, genetically-predicted waist circumference was inversely related to blood adiponectin concentration (β = −0.27 standard unit of log adiponectin per standard unit increase in waist circumference; 95% CI = −0.36, -0.19; *P* = 2*10^−11^), whereas genetically-predicted hip circumference was positively associated with blood adiponectin concentration (β = 0.17 standard unit of log adiponectin per standard unit increase in hip circumference; 95% CI = 0.11, 0.24; *P* = 1*10^−7^).

**Conclusions:**

These results support the hypotheses that there is a complex interplay between body fat distribution and circulating adiponectin concentration, and that whereas obesity-induced hypoadiponectinaemia seems to be primarily attributed to abdominal fat accumulation, gluteofemoral fat accumulation is likely to exert a protective effect.

**Key Messages**

- Circulating adiponectin is substantially reduced among obese individuals, although adiponectin is mainly produced by mature adipocytes.
- Our findings indicate that body fat distribution seems to be a causal determinant of circulating adiponectin and that abdominal and gluteofemoral body fat may have opposite influences regarding modulation of circulating adiponectin.
- Modulation of circulating adiponectin might be a common mediator or biomarker of the detrimental and protective effects of abdominal and gluteofemoral body fat, respectively, in the context of metabolic diseases.

## Introduction

Adiponectin, the most abundant product of adipocytes, circulates in large amounts in the blood (3 to 30 mg/l) and is believed to promote beneficial systemic metabolic effects by interfering with adipogenesis, insulin sensitivity, atherosclerosis and inflammation, as demonstrated in animal models.^1^^,^^2^ Decreased adiponectin concentration is a marker of metabolic/adipose tissue dysfunction and a potential mediator of obesity-related complications.^2^ In humans, higher circulating adiponectin is strongly associated with lower risk of type 2 diabetes,^3^ hepatic dysfunction^4^ and metabolic syndrome,^5^ although recent studies have cast doubt on whether adiponectin concentration is causally related to type 2 diabetes^6^ or coronary heart disease.^7^

Higher adiposity is paradoxically related to a decrease in adiponectin concentration, which seems to be mainly attributed to abdominal visceral fat.^8–13^ However, few previous studies have properly addressed the independent contribution of specific fat depots and none has investigated whether different fat distribution is causally related to blood adiponectin concentration.

Mendelian randomization is a technique that uses genetic variants associated with an exposure, aimed at avoiding potential confounding and reverse causality, to detect whether this exposure is likely to have a causal effect on the outcome of interest, provided that the genetic variant satisfies the assumptions of an instrumental variable (see details in Supplementary Table 1, available as Supplementary data at *IJE* online). Mendelian randomization has several advantages over classical observational studies, as most genetic variants tend to be uncorrelated with conventional epidemiological risk factors. Unlike the exposure itself, genetic variants are fixed at conception and therefore not subject to reverse causation, and genetic variants assessment is subject to relatively little measurement error.^14^ A previous Mendelian randomization study has indicated that high fasting insulin decreases adiponectin concentration.^6^

We aimed to: (i) address whether abdominal fat (visceral, deep subcutaneous and superficial subcutaneous) and gluteofemoral fat are independently associated with blood adiponectin concentration; and (ii) investigate whether abdominal and gluteofemoral fat causally determine blood adiponectin concentration, by using the Mendelian randomization approach.

## Methods

For the conventional association analysis, we used individual-level data from the 1982 Pelotas Birth Cohort (2012 follow-up, when participants were around 30 years old, *n* = 3701 participants) to establish whether abdominal (visceral, deep subcutaneous abdominal and superficial subcutaneous abdominal) and gluteofemoral fat are independently associated with blood adiponectin concentration among young adults.^15^^,^^16^

For the Mendelian randomization analysis, we used summary data from two consortia including multiple studies with genome-wide association scan (GWAS) data to evaluate whether abdominal fat (proxied by waist circumference) and gluteofemoral fat (proxied by hip circumference) are causally related to blood adiponectin concentration: the Genetic Investigation of ANthropometric Traits (GIANT) consortium (*n* = 210 088 participants)^17^ and the ADIPOGen consortium (*n* = 29 347 participants).^18^

### Data sources

#### 1982 Pelotas Birth Cohort: conventional association analysis

Participants were from Pelotas which is a medium-sized southern Brazilian city with nearly 330 000 inhabitants. In 1982, all maternity hospitals in the city were visited daily and 99.2% of the births were identified. Those liveborns whose families lived in the urban area of the city were evaluated and their mothers interviewed (*n* = 5914). Participants have been followed up on several occasions and further details of the study methodology have been described elsewhere.^15^^,^^16^ In 2012, 3701 participants were evaluated who, added to the 325 known to have died, represented a follow-up rate of 68.1%. All phases of the 1982 Pelotas Birth Cohort Study were approved by the Research Ethics Committee of the Federal University of Pelotas, which is affiliated with the Brazilian Federal Medical Council. Written informed consent was obtained from all participating subjects in the 2012 visit.

For body composition and anthropometric measures, abdominal fat depots were measured using the ultrasound machine Toshiba Xario (Toshiba Medical Systems Corp., Tokyo, Japan). Details can be found in previous publication.^19^ Gluteofemoral fat was assessed by dual-energy x-ray absorptiometry (DXA) (Lunar Prodigy Advance—GE, Germany). Details on body composition and anthropometric measures can be found in the Supplementary material (available as Supplementary data at *IJE* online).

For blood adiponectin concentration, serum samples were collected and stored at -70°C. Adiponectin was assayed with the ELISA Quantikine Human Total Adiponectin Immunoassay kit (R&D Systems, Inc., Minneapolis, USA) and SpectraMax 190 microplate spectrophotometer (Molecular Devices Corp, CA, USA). Intra-assay coefficients of variation were estimated based on results from 20 replicates assayed at the same time and under the same conditions. Inter-assay coefficients of variation were estimated based on results from a control sample assayed in every batch. Intra-assay and inter-assay were 6% and 16%, respectively.

Covariates were: sex (male or female), age, African genomic ancestry (%), leisure-time physical activity [inactive (0 min/week), insufficiently active (1 to 149 min/week) or active (≥ 150 min/week)], alcohol drinking (< 1 or ≥ 1 dose/day), smoking (never, ex-smoker, 1 to 10, or ≥ 10 cigarettes/day) and body mass index (BMI; in kg/m^2^). Leisure-time physical activity practice was estimated using the long version of the International Physical Activity Questionnaire (IPAQ).^20^ Genomic ancestry was estimated using 370 539 ancestry informative markers. Details have been published previously^21^ and can be found in Supplementary material.

#### GIANT and ADIPOGen GWAS consortia: Mendelian randomization analysis

The Genetic Investigation of ANthropometric Traits (GIANT) consortium included up to 210 088 individuals of European ancestry from cohorts genotyped with genome-wide single nucleotide polymorphism (SNP) arrays (*n* = 57) or Metabochip (*n* = 44).^17^ Estimates of SNP-waist circumference or SNP-hip circumference association (additive model) were adjusted for age, age^2^, BMI, study-specific covariates and genomic control inflation factor (λ). Summary data for the present study were downloaded from the GIANT consortium website [https://www.broadinstitute.org/collaboration/giant/index.php/GIANT_consortium_data_files].

The ADIPOGen consortium included 29 347 individuals of European ancestry from 16 cohort studies with GWAS data and adiponectin measures.^18^ Estimates of SNP-natural log adiponectin concentration association (additive model) were adjusted for age, sex, BMI, principal components of population stratification, study site (where appropriate), family structure (one family-based study) and genomic control inflation factor (λ). Summary data for the present study were downloaded from ADIPOGen consortium website [https://www.mcgill.ca/genepi/adipogen-consortium]. Details on population characteristics, genotype imputation and quality control criteria for GIANT and ADIPOGen consortia can be found in Supplementary Table 2 (available as Supplementary data at *IJE* online).

### Data analysis

#### 1982 Pelotas Birth Cohort: conventional association analysis

Adiponectin was log-transformed prior to analyses owing to positive skewness. Log-adiponectin and visceral, deep and superficial subcutaneous abdominal fat thickness (cm) and gluteofemoral fat mass (kg) were standardized for each sex. We used unadjusted and adjusted linear regression models to estimate the association of the fat depots with adiponectin concentration. Adjusted models were controlled for genomic ancestry, smoking status, alcohol intake, leisure-time physical activity and other fat depots. The correlation across fat depots and BMI was estimated by Pearson’s correlation coefficient. To explore nonlinear relations between fat depots and adiponectin concentration, we used two-degree fractional polynomial (FP) models. FP models were fitted separately for each fat depot and for each sex, adjusting for study covariates (eight models in total). The best-fitting adjusted FP model was compared with the corresponding adjusted linear model using likelihood ratio (LR) test. Departures from linearity were assessed by *P*-values from LR testing after Bonferroni correction (Bonferroni corrected *P*-values = 0.05/8 = 0.00625). All analyses were conducted separately according to sex, excluding pregnant women (*n* = 73) and were based on complete cases (no missing information in study covariates).

Sensitivity analysis: we investigated whether observations were missing completely at random (MCAR) by testing the association of our complete case analysis indicator with all study covariates. Missing values were imputed with multivariate imputation using chained equations (MICE) for 20 complete datasets with 10 iterations each. Multiple imputation was performed separately for each sex. All study variables were included in the model for imputing missing variables (African genomic ancestry, leisure-time physical activity, alcohol drinking, smoking, BMI, adiponectin concentration and fat depots). The same unadjusted and adjusted linear regression models previously described were fitted using the imputed dataset. Coefficients and standard errors for the variability between imputations were combined according to Rubin’s rules. ^22^

#### GIANT and ADIPOGen GWAS consortia: Mendelian randomization analysis

All SNPs associated with waist or hip circumference in the GIANT consortium at GWAS threshold *P*-value < 5*10^−8^ were selected, and variants in linkage disequilibrium (R^2 ^< 0.05) were removed using 1000 Genomes reference population and SNP Annotation and Proxy Search (SNAP) tool.^23^ This resulted in 64 SNPs for waist circumference and 83 SNPs for hip circumference. After the exclusion of eight overlapping variants, 56 SNPs and 75 SNPs were selected as instrumental variables for waist and hip circumference, respectively, in the Mendelian randomization analysis (Supplementary Tables 3 and 4, available as Supplementary data at *IJE* online). We estimated that the 56 SNPs used as instruments for waist circumference explain around 1.2% of waist circumference phenotypic variance, and the 75 SNPs used as instruments for hip circumference explain around 2.0% of hip circumference phenotypic variance (details on proportion of phenotypic variance explained estimation and power calculations can be found in Supplementary methods). Data on the association of SNPs with (i) waist or hip circumference and (ii) blood adiponectin concentration were combined using the inverse-variance weighted (IVW) method, described by Burgess *et al.*^24^ Two main models were used in Mendelian randomization analysis: (I) unadjusted model^24^; (II) adjusted model, in which a multivariate IVW method was used to adjust the effect of waist circumference on adiponectin concentration for hip circumference and vice versa^25^ (see Supplementary material for details on IVW method). To evaluate whether genetically increased adiponectin concentration could influence fat distribution, we selected, as instrumental variables for adiponectin concentration, four SNPs (rs6810075, rs16861209, rs17366568, rs3774261) within *ADIPOQ* gene (± 25 kb) (Supplementary Table 5, available as Supplementary data at *IJE* online). These SNPs have previously been selected by Dastani *et al.*(2013)^26^ by linkage disequilibrium (LD) pruning of 145 genome-wide significant SNPs in the ADIPOGen consortium,^27^ retaining SNPs that explained most variance in adiponectin concentration in each LD block [LD threshold: R^2 ^< 0.05 in HapMap CEU population (Utah residents with Northern and Western European ancestry)]. We estimate that these four SNPs explain around 4.0% of the variance in adiponectin concentration (details on proportion of phenotypic variance explained estimation and power calculations can be found in Supplementary methods). Mendelian randomization results for the effect of adiponectin concentration on waist and hip circumference were also estimated by the IVW method.

Sensitivity analyses: to assess the validity of causal inference from our main Mendelian randomization findings, we conducted a series of sensitivity analyses based on two stages.^28^ In stage one, we investigated the presence of heterogeneity and asymmetry in causal estimates from each genetic variant using standard methods from meta-analysis literature. Heterogeneity was assessed by visually inspecting the forest plot of per SNP Wald ratio estimate and by estimating I^2^ (and respective 95% CI), a measure of the relative size of between-study variation and within-study error, and *P*-value for heterogeneity for Cochran’s Q test.^29^^,^^30^ Asymmetry was evaluated using funnel plot and Egger’s test.^28^^,^^31^ Assuming that all valid instrumental variables identify the same causal parameter, substantial heterogeneity would be suggestive of pleiotropic SNPs and asymmetry could indicate directional (unbalanced) pleiotropy, meaning that the overall causal estimate is biased.

In stage two, we used other Mendelian randomization estimators based on a less stringent set of assumptions than a conventional Mendelian randomization analysis (IVW method). Two methods were used: the penalized weighted median estimator^32^ and the Mendelian randomization (MR)-Egger method.^33^ The weighted median estimator gives consistent estimates even if up to 50% of weight in the analysis is from invalid instrumental variables and downweights (penalizes) the contribution of heterogeneous variants. The MR-Egger method gives consistent estimates even if all the genetic variants are invalid instruments, provided that the InSIDE (Instrument Strength Independent of Direct Effect) assumption holds, which requires that there is no correlation between SNP-exposure association and direct effects of SNP on outcome.^33^ Bootstrapping was used to derive corrected 95% confidence intervals for both penalized weighted median and the MR-Egger estimates.^32^^,^^33^ See Supplementary methods (available as Supplementary data at *IJE* online) for a detailed description of the penalized weighted median estimator and MR-Egger method.

## Results

### 

#### 1982 Pelotas Birth Cohort: conventional association analysis

Main results: participants’ characteristics are described in Table 1; 2743 individuals (1315 males and 1428 females) had complete information on all study variables [mean age 30·2 years; standard deviation (SD): 0·3]. Median blood adiponectin concentration was 6237 ng/ml (interquartile interval: 4163, 8979) in men and 10 067 ng/ml (interquartile interval: 7002, 14 282) in women. The association of adiponectin concentration and fat depots with study covariates (African ancestry and lifestyle characteristics) are displayed in Supplementary Table 6.

**Table 1:** Participants’ characteristics at 30 years of age. 1982 Pelotas Birth Cohort, 2012

	Male		Female		Total	
**Ancestry & lifestyle variables, *n* and %**						
African ancestry (%)						
0.00–4.59	449	34.1	472	33.1	921	33.6
4.60–10.99	430	32.7	482	33.8	912	33.2
11.00–87.91	436	33.2	474	33.2	910	33.2
Leisure-time physical activity						
Inactive	440	33.5	890	62.3	1330	48.5
Insufficiently active	367	27.9	237	16.6	604	22.0
Active	508	38.6	301	21.1	809	29.5
Smoking						
Never smoker	737	56.0	853	59.7	1590	58.0
Ex-smoker	232	17.6	262	18.3	494	18.0
1–9 cigarettes/day	104	7.9	127	8.9	231	8.4
≥ 10 cigarettes/day	242	18.4	186	13.0	428	15.6
Alcohol drinking						
< 1 dose/day	481	36.6	917	64.2	1398	51.0
≥ 1 dose/day	834	63.4	511	35.8	1345	49.0
**Anthropometry, body composition & adiponectin levels, mean and SD**						
Body mass index (kg/m^2^)	26.6	4.4	26.7	5.8	26.6	5.1
Total fat (kg)	20.6	9.9	27.9	11.4	24.4	11.3
Fat depots:						
Visceral (cm)	6.8	1.9	4.9	1.6	5.8	2.0
Deep subcutaneous abdominal (cm)	1.2	0.7	1.5	0.8	1.4	0.8
Superficial subcutaneous abdominal (cm)	0.7	0.3	1.0	0.5	0.9	0.5
Gluteofemoral (kg)	3.7	1.6	5.4	1.9	4.6	2.0
Adiponectin (ng/ml)^a^	7208	4411	11290	6033	9333	5694
*n* total	1315		1428		2743	

Data from the 2012 follow-up of the 1982 Pelotas Birth Cohort. SD, standard deviation.

^a^Median blood adiponectin concentration was 6237 ng/ml (interquartile range: 4163, 8979) in men and 10 067 ng/ml (interquartile range: 7002, 14 282) in women.

**Figure 1:**
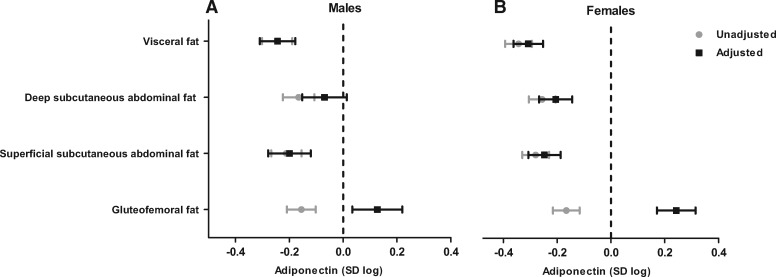
Mean difference (95% CI) in standardized log adiponectin concentration per unit increase in standardized fat depots for males (A) and females (B). Unadjusted models estimates are represented by grey dots and adjusted models by black squares. Adjusted models included genomic ancestry, smoking status, alcohol intake and other fat depots. SD, standard deviation. Data from the 2012 follow-up of the 1982 Pelotas Birth Cohort.

**Figure 2:**
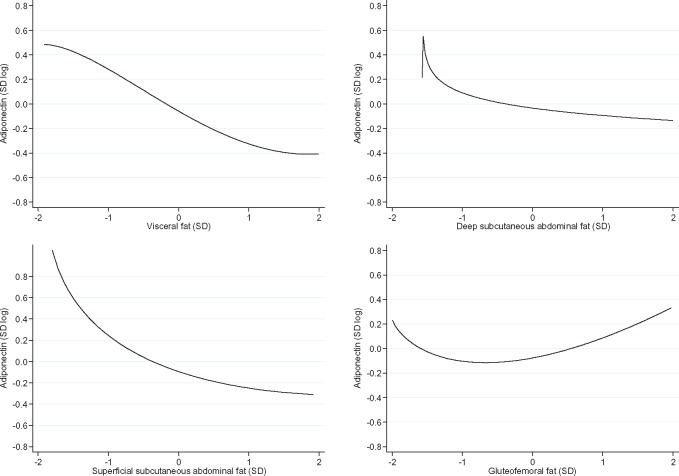
Dose-response relation between fat depots and adiponectin concentration in males. (A) Visceral fat (*P* for nonlinear trend = 0.003); (B) deep subcutaneous abdominal fat (*P* for nonlinear trend = 0.121); (C) superficial subcutaneous abdominal fat (*P* for nonlinear trend = 5*10^−6^); (D) gluteofemoral fat (*P* for nonlinear trend = 3*10^−4^). SD, standard deviation. Data from the 2012 follow-up of the 1982 Pelotas Birth Cohort.

**Figure 3:**
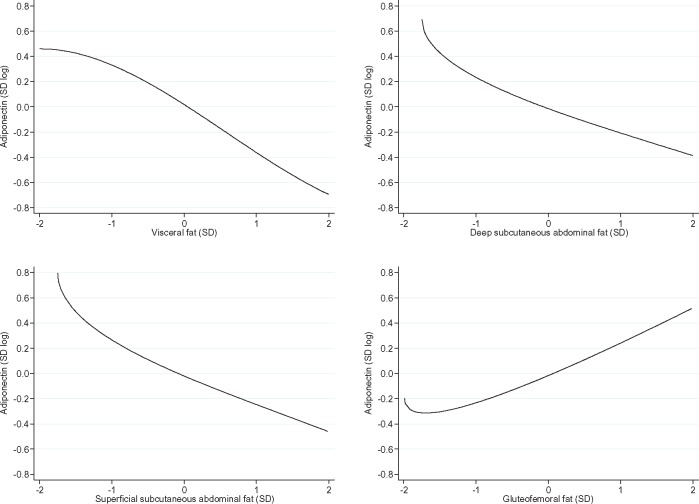
Dose-response relation between fat depots and adiponectin concentration in females. (A) Visceral fat (*P* for nonlinear trend = 0.006); (B) deep subcutaneous abdominal fat (*P* for nonlinear trend = 0.105); (C) superficial subcutaneous abdominal fat (*P* for nonlinear trend = 0.058); (D) gluteofemoral fat (*P* for nonlinear trend = 0.037). SD, standard deviation. Data from the 2012 follow-up of the 1982 Pelotas Birth Cohort.

Subcutaneous fat depots (deep abdominal, superficial abdominal and gluteofemoral) were moderately to highly correlated among each other (r = 0.46, 0.71) and moderately correlated with visceral fat (r = 0.30, 0.53) (Supplementary Table 7, available as Supplementary data at *IJE* online). In unadjusted linear models, all fat depots were strongly and negatively associated with blood adiponectin concentration (Figure 1). After adjusting linear models for other fat depots and study covariates, the association between gluteofemoral fat and adiponectin concentration became positive (men: β = 0.13 standard unit of log adiponectin per standard unit increase in gluteofemoral fat; 95% CI = 0.03, 0.22; *P* = 0.008; women: β = 0.24; 95% CI = 0.17, 0.31; *P* = 7*10^−11^). The association of adiponectin concentration with deep subcutaneous abdominal fat was attenuated in the adjusted models especially among men (men: β = −0.07 standard unit of log adiponectin per standard unit increase in deep subcutaneous abdominal fat; 95% CI = −0.15, 0.01; *P* = 0.10; women: β = −0.21; 95% CI = −0.27, -0.14; *P* = 8*10^−11^), and remained similar for visceral (men: β = −0.24 standard unit of log adiponectin per standard unit increase in visceral fat; 95% CI = −0.31, -0.18; *P* = 8*10^−13^; women: β = −0.31; 95% CI = −0.36, -0.25; *P* = 7*10^−27^) and superficial subcutaneous abdominal fat (men: β = −0.20 standard unit of log adiponectin per standard unit increase in superficial subcutaneous abdominal fat; 95% CI = −0.28, −0.12; p = 8*10^−7^; women: β = −0.25; 95% CI = −0.31, −0.19; *P* = 6*10^−16^) (Figure 1). Among men, there was a monotonic but nonlinear trend in the relation of adiponectin concentration with visceral and superficial subcutaneous abdominal fat (*P*-value for nonlinear trend = 0.003 and 5*10^−6^, respectively) and a ‘U’-shaped curve in the association of adiponectin concentration and gluteofemoral fat (*P*-value for nonlinear trend = 3*10^−4^) (Figure 2). Among women, fat depots were associated with adiponectin in a linear fashion, except in the case of visceral fat (*P*-value for nonlinear trend = 0.006) (Figure 3).

Sensitivity analysis: overall, missingness was not associated with study variables in females and was associated with BMI, visceral fat, deep and superficial subcutaneous abdominal fat in males (Supplementary Table 8, available as Supplementary data at *IJE* online). Overall, results from complete case (Figure 1) and imputed models (Table 2) were similar.

**Table 2:** Mean difference (95% CI) in log adiponectin concentration per unit increase in fat depots for males (A) and females (B) after multiple imputation

	Males (*n* = 1787)							Females (*n* = 1841)						
	Crude				Adjusted			Crude				Adjusted		
Variables	*n* missing	β	95% CI	*P*-value	β	95% CI	*P*-value	*n* missing	β	95% CI	*P*-value	β	95% CI	*P*-value
VAT	63	−0.24	(−0.29, −0.20)	4*10^−25^	−0.25	(−0.31, −0.20)	4*10^−18^	76	−0.35	(−0.40, −0.31)	3*10^−56^	−0.31	(−0.35, −0.26)	4*10^−34^
Deep SCAT	48	−0.14	(−0.19, −0.09)	1*10^−09^	−0.06	(−0.13, 0.00)	3*10^−02^	71	−0.25	(−0.30, −0.21)	3*10^−28^	−0.19	(−0.25, −0.13)	4*10^−11^
Superficial SCAT	48	−0.17	(−0.21, −0.12)	1*10^−12^	−0.13	(−0.19, -0.06)	1*10^−04^	71	−0.29	(−0.33, −0.25)	3*10^−37^	−0.24	(−0.29, −0.19)	5*10^−19^
GFAT	112	−0.14	(−0.19, −0.10)	1*10^−11^	0.10	(0.02, 0.18)	2*10^−02^	85	−0.18	(−0.23, -0.14)	9*10^−17^	0.21	(0.14, 0.27)	7*10^−10^

Adjusted models included age, genomic ancestry, smoking status, alcohol intake, and other fat depots. Data from the 2012 follow-up of the 1982 Pelotas Birth Cohort (excluding 73 pregnant women). GFAT; gluteofemoral adipose tissue, SCAT; subcutaneous adipose tissue, VAT; visceral adipose tissue.

#### GIANT and ADIPOGen GWAS consortia: Mendelian randomization analysis

**Figure 4:**
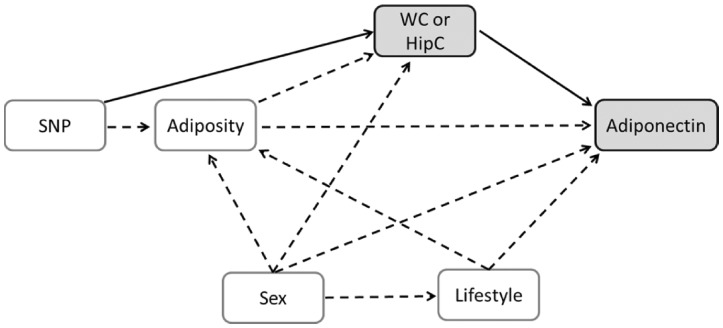
Causal diagram representing assumed causal relationships in the Mendelian randomization analysis. The solid lines represent the relationships being tested (i.e. effect of WC or HipC on adiponectin concentration using genetic instruments). WC, waist circumference; HipC, hip circumference; SNP, single nucleotide polymorphisms.

**Figure 5:**
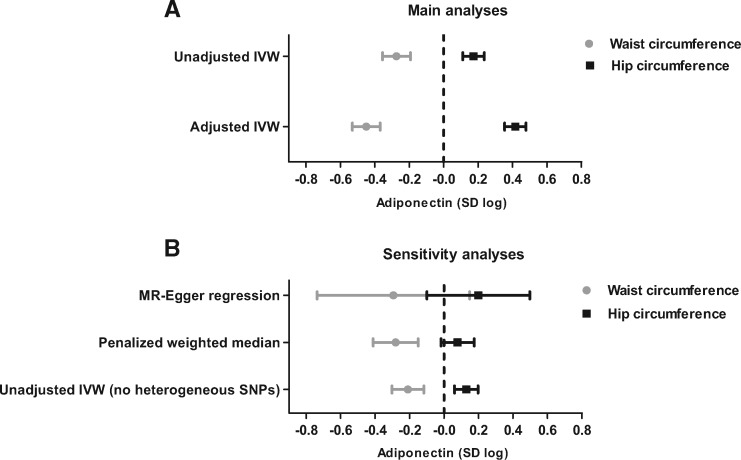
Main (A) and sensitivity (B) Mendelian randomization analyses of the mean difference (95% CI) in standardized log adiponectin concentration per unit increase in standardized waist (grey dots) or hip circumference (black squares). Data from GIANT (*n* = up to 210 088 individuals) and ADIPOGen (*n* = 29 347 individuals) consortia. Adjusted IVW model: IVW method adjusted for hip circumference (in waist circumference model) or waist circumference (in hip circumference model). Waist and hip circumference were adjusted by body mass index before analysis. IVW method, inverse variance method; MR, Mendelian randomization.

We used summary data from GIANT and ADIPOGen consortia to perform a two-sample Mendelian randomization analysis aimed at investigating the causal influence of accumulating abdominal (proxied by waist circumference) or gluteofemoral (proxied by hip circumference) fat on adiponectin concentration (Figure 4). In unadjusted IVW models, genetically predicted waist circumference was inversely related to blood adiponectin concentration (β = −0.27 standard unit of log adiponectin per standard unit increase in waist circumference; 95% CI = −0.36, -0.19; *P* = 2*10^−11^), whereas genetically predicted hip circumference was positively associated with blood adiponectin concentration (β = 0.17 standard unit of log adiponectin per standard unit increase in hip circumference; 95% CI = 0.11, 0.24; *P* = 1*10^−7^). In the adjusted IVW models, adjusting waist circumference models for hip circumference and vice versa produced larger effect size estimates (waist circumference: β = −0.45; 95% CI = −0.53, -0.37; *P* = 1*10^−27^; hip circumference: β = 0.42; 95% CI = 0.35, 0.48; *P* = 1*10^−38^) (Figure 5). We also performed a reverse Mendelian randomization analysis to test whether genetically predicted adiponectin concentration could influence fat distribution; our findings did not support a role of adiponectin concentration in either waist (β = −0.01 standard unit per standard unit increase in log adiponectin; 95% CI = −0.03, 0.01; *P* = 0.23) or hip circumference (β = 0.00 standard unit per standard unit increase in log adiponectin; 95% CI = −0.03, 0.02; *P* = 0.39).

Substantial heterogeneity was identified among Mendelian randomization estimates from genetic variants used as instrumental variables for waist (I^2 ^= 72%; 95% CI: 66, 77%; *P*-value for heterogeneity = 1*10^−17^) and hip (I^2 ^= 46%; 95% CI: 37, 54%; *P*-value for heterogeneity = 9*10^−6^) circumference (Supplementary Figures 1 and 2, available as Supplementary data at *IJE* online). However, there was no strong evidence of directional pleiotropy as evidenced by the absence of substantial asymmetry in funnel plots and by the *P*-value for the Egger test (*P* = 0.45 for waist and *P* = 0.51 for hip circumference) (Supplementary Figure 3, available as Supplementary data at *IJE* online).

**Figure 6:**
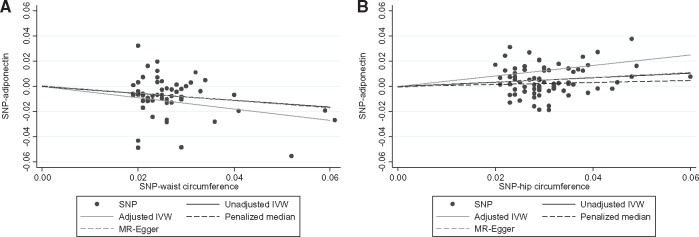
Mean difference in standardized log adiponectin concentration according to genetically increased standardized waist (A) or hip (B) circumference. Data from GIANT (*n* = up to 210 088 individuals) and ADIPOGen (*n* = 29 347 individuals) consortia. Each data point represents betas for SNP-adiponectin (Y axis) and SNP-waist or hip circumference (X axis) association. Predicted values for the main analyses are represented by the full black line (unadjusted IVW method) and full grey line (adjusted IVW method). Predicted values for the sensitivity analyses are represented by the dashed black line (weighted median estimator) and dashed grey line (MR-Egger method). IVW method, inverse-variance weighted method; MR-Egger method, Mendelian randomization-Egger method.

In the sensitivity analysis, we used other Mendelian randomization methods (MR-Egger regression method and penalized weighted median estimator) to investigate the potential impact of invalid instruments on our Mendelian randomization estimates using the IVW method. The penalized weighted median estimator indicated that each increase in standardized waist or hip circumference was related to a variation of -0.28 (95% CI: -0.41, -0.15; *P* = 1*10^−5^) and 0.08 (95% CI: 0.02, 0.17; *P* = 0.11), respectively, in standardized log adiponectin concentration (Figures 5 and 6). The MR-Egger method predicted that each unit increase in standardized waist or hip circumference was related to a variation of -0.29 (95% CI: -0.74, 0.15; *P* = 0.10) and 0.20 (95% CI: -0.10; 0.50; *P* = 0.17), respectively, in standardized log adiponectin concentration with no evidence of directional pleiotropy (intercept for waist circumference = 0.00; 95% CI: -0.01, 0.01; *P* = 0.40; intercept for hip circumference = 0.00; 95% CI: -0.01, 0.01; *P* = 0.43) (Figure 6). We also repeated the unadjusted IVW method after removing heterogeneous genetic variants (12 SNPs from the waist and 13 SNPs from the hip circumference instrument), defined as those with Q statistics for IVW estimates above 3.84, considering a chi-square distribution with one degree of freedom, and results were similar (waist circumference: β = −0.21; 95% CI: −0.30, −0.12; *P* = 4*10^−6^; hip circumference: β = 0.13; 95% CI: 0.06, 0.20; *P* = 4*10^−4^).

## Discussion

Our findings reinforce previous evidence for a complex interplay between body fat distribution and circulating adiponectin concentration.^8–13^ The present results advance previous studies by showing that body fat distribution seems to be a causal determinant of circulating adiponectin and that abdominal and gluteofemoral fat may have opposite influences regarding modulation of circulating adiponectin. In contrast, our results suggest that circulating adiponectin concentration is unlikely to influence body fat distribution.

Low adiponectin concentration has been previously reported to be associated with increased abdominal visceral fat mass.^8–13^ We observed that abdominal fat, regardless of visceral or subcutaneous location, was negatively correlated with adiponectin. In addition, findings from the Mendelian randomization analysis are supportive of the hypothesis that abdominal fat accumulation lowers adiponectin concentration, corroborating the hypothesis that obesity-induced hypoadiponectinaemia can be primarily attributed to the expansion of abdominal fat mass. Interestingly, estimates from both conventional regression and Mendelian randomization were of similar magnitude, despite differences in characteristics of the study populations (e.g. ancestry and age distribution) and in length of exposure time.

We also observed that gluteofemoral fat was positively associated with adiponectin concentration, in agreement with previous results.^12^^,^^13^^,^^34^^,^^35^ This association only became apparent in conventional regression analysis after accounting for abdominal fat, and became stronger in Mendelian randomization analysis after accounting for waist circumference. Our findings that individuals genetically predisposed to gluteofemoral fat accumulation have higher adiponectin concentration are supportive of the increasingly acknowledged protective effect of gluteofemoral fat in the context of metabolic diseases. It is hypothesized that peripheral subcutaneous compartments act as lipid-buffering tissues, protecting several organs/tissues from ectopic fat deposition, and that expansion of gluteofemoral fat mass could prevent the development of metabolic dysfunction when facing energy surplus.^36^^,^^37^

Intrinsic functional differences are likely to explain the opposing modulation of abdominal visceral and gluteofemoral fat on adiponectin concentration. Adiponectin production by cultured adipocytes from the visceral fat compartment (omentum) is affected by both insulin and insulin-sensitizing drugs (e.g. rosiglitazone), whereas subcutaneous fat seems to be nonresponsive.^9^ Glucocorticoids, prolactin and growth hormone are also known to modulate adiponectin production,^38^^,^^39^ but it is not clear how specific fat compartments might respond differently to these hormones. Depot-specific modulation of adiponectin concentration may also be related to differences in tissue local microenvironment, especially with reference to adipocytokine secretion pattern; for example, tumour necrosis factor (TNF)-α expression, an inhibitor of adiponectin production, is increased in abdominal visceral fat expansion.^40^

Overproduction of adiponectin in animal models can induce substantial expansion of subcutaneous fat depots,^41^ which is consistent with the capacity of adiponectin to activate peroxisome proliferator activator receptor (PPAR)-γ, a key transcription factor of adipogenesis. In humans, PPAR-y agonists, such as thiazolidinediones, increase fat mass particularly the subcutaneous compartment.^42^ This raises the question of whether adiponectin is directly playing a protective role against ectopic fat deposition by promoting the expansion of gluteofemoral fat mass. However, our findings are not supportive of the hypothesis that genetically increased adiponectin concentration influences either abdominal or gluteofemoral fat accumulation.

This is one of the largest studies to address the independent contribution of several fat depots to adiponectinaemia, using detailed data on body composition. We have followed a rigorous analysis plan by accounting for multiple important confounders, exploring nonlinear associations between exposure and outcome and conducting multiple imputation to investigate the presence of bias from the complete case analysis. This is also the first study to use Mendelian randomization to assess the causal relations between body fat distribution and adiponectin concentration. In our Mendelian randomization analysis, we established a systematic approach to selecting our instrumental variables and conducted a range of sensitivity analyses to assess the robustness of our findings.

The main limitation in our Mendelian randomization analysis is the use of multiple genetic variants as instrumental variables, for most of which there is no clear biological understanding on how they influence fat distribution. Therefore, it is possible that at least some variants violate the instrumental variables assumption due to horizontal pleiotropy, which could be the case if some variants affect adiponectin concentration independently of their effect on the exposure of interest (i.e. waist or hip circumference). Although we cannot discard the possibility of horizontal pleiotropy biasing our results, we did show that this is unlikely since there was no evidence of directional pleiotropy and Mendelian randomization estimates from different methods (with different assumptions) were generally consistent with findings from the IVW method. Another potential limitation is the adjustment of SNP-waist or SNP-hip circumference models for BMI, a proxy of whole body adiposity, which could introduce collider bias in the Mendelian randomization analysis as illustrated by Figure 4. However, it should be emphasized that: (i) such bias should act in the same direction for both waist and hip circumference and, therefore, could not explain the opposing effects of waist and hip circumference with regards to adiponectin concentration; and (ii) had we not adjusted for whole body adiposity (proxied by BMI), we would not be able to disentangle the effects of waist from hip circumference and vice versa, as instruments for both traits would be highly correlated to whole-body adiposity. A third limitation in the Mendelian randomization analysis is the use of summary data, which precluded us from investigating sex-specific and nonlinear effects.

In summary, our findings suggest that body fat distribution is a causal determinant of adiponectin concentration, whereas adiponectin concentration does not seem to influence abdominal or gluteofemoral fat accumulation. Our results add to the understanding of the complex metabolic regulation by adipose tissue, and indicate that modulation of adiponectin concentration might be a common marker of the detrimental and protective effects of abdominal and gluteofemoral fat, respectively, in the context of metabolic diseases.

## Supplementary Data

Supplementary data are available at *IJE* online.

## Funding

The study ‘Pelotas Birth Cohort, 1982’ is conducted by Postgraduate Program in Epidemiology at Universidade Federal de Pelotas with the collaboration of the Brazilian Public Health Association (ABRASCO). From 2004 to 2013, the Wellcome Trust supported the 1982 birth cohort study. The International Development Research Center, World Health Organization, Overseas Development Administration, European Union, National Support Program for Centers of Excellence (PRONEX), the Brazilian National Research Council (CNPq) and the Brazilian Ministry of Health supported previous phases of the study. M.C.B. receives financial support from the Brazilian National Research Council (CNPq) [144749/2014‐9, 201498/2014‐6 (Science Without Borders Program), and 163291/2015‐2] and Coordenação de Aperfeiçoamento de Pessoal de Nível Superior (CAPES). K.K.O. is supported by the Medical Research Council [Unit Programme numbers MC_UU_12015/1 and MC_UU_12015/2]. The funders had no role in study design, data collection and analysis, decision to publish or preparation of the manuscript.

## Supplementary Material

Supplementary Data

## References

[1] Yamauchi T, Nio Y, Maki T Targeted disruption of AdipoR1 and AdipoR2 causes abrogation of adiponectin binding and metabolic actions. Nat Med 2007;13:332–39. 17268472 10.1038/nm1557

[2] Turer AT, Scherer PE Adiponectin: mechanistic insights and clinical implications. Diabetologia 2012;55:2319–26. 22688349 10.1007/s00125-012-2598-x

[3] Li S, Shin HJ, Ding EL, van Dam RM Adiponectin Levels and Risk of Type 2 Diabetes: A Systematic Review and Meta-analysis. JAMA 2009;302:179–88. 19584347 10.1001/jama.2009.976

[4] Polyzos SA, Toulis KA, Goulis DG, Zavos C, Kountouras J Serum total adiponectin in nonalcoholic fatty liver disease: a systematic review and meta-analysis. Metabolism 2011;60:313–26. 21040935 10.1016/j.metabol.2010.09.003

[5] Matsuzawa Y, Funahashi T, Kihara S, Shimomura I Adiponectin and metabolic syndrome. Arterioscler Thromb Vasc Biol 2004;24:29–33. 14551151 10.1161/01.ATV.0000099786.99623.EF

[6] Yaghootkar H, Lamina C, Scott RA Mendelian randomization studies do not support a causal role for reduced circulating adiponectin levels in insulin resistance and type 2 diabetes. Diabetes 2013;62:3589–98. 23835345 10.2337/db13-0128 PMC3781444

[7] Borges MC, Lawlor DA, de Oliveira C, White J, Horta BL, Barros AJ Role of Adiponectin in Coronary Heart Disease Risk: A Mendelian Randomization Study. Circ Res 2016;119:491–99. 27252388 10.1161/CIRCRESAHA.116.308716 PMC4959825

[8] Drolet R, Bélanger C, Fortier M Fat depot-specific impact of visceral obesity on adipocyte adiponectin release in women. Obesity (Silver Spring) 2009;17:424–30. 19219061 10.1038/oby.2008.555

[9] Motoshima H, Wu X, Sinha MK Differential regulation of adiponectin secretion from cultured human omental and subcutaneous adipocytes: effects of insulin and rosiglitazone. J Clin Endocrinol Metab 2002;87:5662–67. 12466369 10.1210/jc.2002-020635

[10] Cnop M, Havel PJ, Utzschneider KM Relationship of adiponectin to body fat distribution, insulin sensitivity and plasma lipoproteins: evidence for independent roles of age and sex. Diabetologia 2003;46:459–69. 12687327 10.1007/s00125-003-1074-z

[11] Park KG, Park KS, Kim MJ Relationship between serum adiponectin and leptin concentrations and body fat distribution. Diabetes Res Clin Pract 2004;63:135–42. 14739054 10.1016/j.diabres.2003.09.010

[12] Snijder MB, Flyvbjerg A, Stehouwer CD Relationship of adiposity with arterial stiffness as mediated by adiponectin in older men and women: the Hoorn Study. Eur J Endocrinol 2009;160:387–95. 19095778 10.1530/EJE-08-0817

[13] Hanley AJ, Bowden D, Wagenknecht LE Associations of adiponectin with body fat distribution and insulin sensitivity in nondiabetic Hispanics and African-Americans. J Clin Endocrinol Metab 2007;92:2665–71. 17426091 10.1210/jc.2006-2614

[14] Davey Smith G, Hemani G Mendelian randomization: genetic anchors for causal inference in epidemiological studies. Hum Mol Genet 2014;23:R89–98. 25064373 10.1093/hmg/ddu328 PMC4170722

[15] Victora CG, Barros FC Cohort profile: The 1982 Pelotas (Brazil) birth cohort study. Int J Epidemiol 2006;35:237–42. 16373375 10.1093/ije/dyi290

[16] Horta BL, Gigante DP, Goncalves H Cohort Profile Update: The 1982 Pelotas (Brazil) Birth Cohort Study. Int J Epidemiol 2015;44**:** 441. 25733577 10.1093/ije/dyv017 PMC4469796

[17] Shungin D, Winkler TW, Croteau-Chonka DC New genetic loci link adipose and insulin biology to body fat distribution. Nature 2015;518:187–96. 25673412 10.1038/nature14132 PMC4338562

[18] Dastani Z, Hivert MF, Timpson N Novel loci for adiponectin levels and their influence on type 2 diabetes and metabolic traits: a multi-ethnic meta-analysis of 45,891 individuals. PLoS Genet 2012;8:e1002607. 22479202 10.1371/journal.pgen.1002607 PMC3315470

[19] Araujo de Franca GV, Lucia Rolfe E, Horta BL Associations of birth weight, linear growth and relative weight gain throughout life with abdominal fat depots in adulthood: the 1982 Pelotas (Brazil) birth cohort study. Int J Obes (Lond) 2016;40**:** 14–21. 26395747 10.1038/ijo.2015.192 PMC4722236

[20] Craig CL, Marshall AL, Sjostrom M International physical activity questionnaire: 12-country reliability and validity. Med Sci Sports Exerc 2003;35:1381–95. 12900694 10.1249/01.MSS.0000078924.61453.FB

[21] Lima-Costa MF, Rodrigues LC, Barreto ML Genomic ancestry and ethnoracial self-classification based on 5,871 community-dwelling Brazilians (The Epigen Initiative). Sci Rep 2015;5:9812. 25913126 10.1038/srep09812 PMC5386196

[22] Rubin DB Multiple Imputation for Nonresponse in Surveys. New York, NY: Wiley, 1987.

[23] Johnson AD, Handsaker RE, Pulit SL, Nizzari MM, O’Donnell CJ, de Bakker PI SNAP: a web-based tool for identification and annotation of proxy SNPs using HapMap. Bioinformatics 2008;24:2938–39. 18974171 10.1093/bioinformatics/btn564 PMC2720775

[24] Burgess S, Butterworth A, Thompson SG Mendelian randomization analysis with multiple genetic variants using summarized data. Genet Epidemiol 2013;37:658–65. 24114802 10.1002/gepi.21758 PMC4377079

[25] Burgess S, Dudbridge F, Thompson SG Re:’Multivariable Mendelian randomization: the use of pleiotropic genetic variants to estimate causal effects’. Am J Epidemiol 2015;181:290–91. 25660081 10.1093/aje/kwv017

[26] Dastani Z, Johnson T, Kronenberg F The shared allelic architecture of adiponectin levels and coronary artery disease. Atherosclerosis 2013;229:145–48. 23664276 10.1016/j.atherosclerosis.2013.03.034 PMC6139652

[27] Dastani Z, Hivert MF, Timpson N Novel loci for adiponectin levels and their influence on type 2 diabetes and metabolic traits: a multi-ethnic meta-analysis of 45,891 individuals. PLoS Genet 2012;8:e1002607. 22479202 10.1371/journal.pgen.1002607 PMC3315470

[28] Burgess S, Bowden J, Fall T, Ingelsson E, Thompson SG Sensitivity analyses for robust causal inference from Mendelian randomization analyses with multiple genetic variants. Epidemiology 2017;28**:** 30–42. 27749700 10.1097/EDE.0000000000000559 PMC5133381

[29] Del Greco MF, Minelli C, Sheehan NA, Thompson JR Detecting pleiotropy in Mendelian randomisation studies with summary data and a continuous outcome. Stat Med 2015;34:2926–40. 25950993 10.1002/sim.6522

[30] Higgins JP, Thompson SG Quantifying heterogeneity in a meta-analysis. Stat Med 2002;21:1539–58. 12111919 10.1002/sim.1186

[31] Egger M, Davey Smith G, Schneider M, Minder C Bias in meta-analysis detected by a simple, graphical test. BMJ 1997;315:629–34. 9310563 10.1136/bmj.315.7109.629 PMC2127453

[32] Bowden J, Davey Smith G, Haycock PC, Burgess S Consistent estimation in Mendelian randomization with some invalid instruments using a weighted median estimator. Genet Epidemiol 2016;40**:** 304–14. 27061298 10.1002/gepi.21965 PMC4849733

[33] Bowden J, Davey Smith G, Burgess S Mendelian randomization with invalid instruments: effect estimation and bias detection through Egger regression. Int J Epidemiol 2015;44:512–25. 26050253 10.1093/ije/dyv080 PMC4469799

[34] Turer AT, Khera A, Ayers CR Adipose tissue mass and location affect circulating adiponectin levels. Diabetologia 2011;54:2515–24. 21779869 10.1007/s00125-011-2252-z PMC4090928

[35] Buemann B, Sørensen TI, Pedersen O Lower-body fat mass as an independent marker of insulin sensitivity - the role of adiponectin. Int J Obes (Lond) 2005;29:624–31. 15824752 10.1038/sj.ijo.0802929

[36] Tchernof A, Despres JP Pathophysiology of human visceral obesity: an update. Physiol Rev 2013;93:359–404. 23303913 10.1152/physrev.00033.2011

[37] Karpe F, Pinnick KE Biology of upper-body and lower-body adipose tissue - link to whole-body phenotypes. Nat Rev Endocrinol 2015;11:90–100. 25365922 10.1038/nrendo.2014.185

[38] Fasshauer M, Klein J, Neumann S, Eszlinger M, Paschke R Hormonal regulation of adiponectin gene expression in 3T3-L1 adipocytes. Biochem Biophys Res Commun 2002;290:1084–89. 11798186 10.1006/bbrc.2001.6307

[39] Swarbrick MM, Havel PJ Physiological, pharmacological, and nutritional regulation of circulating adiponectin concentrations in humans. Metab Syndr Relat Disord 2008;6:87–102. 18510434 10.1089/met.2007.0029 PMC3190268

[40] Van Gaal LF, Mertens IL, De Block CE Mechanisms linking obesity with cardiovascular disease. Nature 2006;444:875–80. 17167476 10.1038/nature05487

[41] Kim JY, van de Wall E, Laplante M Obesity-associated improvements in metabolic profile through expansion of adipose tissue. J Clin Invest 2007;117:2621–37. 17717599 10.1172/JCI31021 PMC1950456

[42] Yang X, Smith U Adipose tissue distribution and risk of metabolic disease: does thiazolidinedione-induced adipose tissue redistribution provide a clue to the answer?. Diabetologia 2007;50:1127–39. 17393135 10.1007/s00125-007-0640-1

