# Supplementary material for: Obesity-induced hypoadiponectinaemia: the opposite influences of central and peripheral fat compartments
Source: Int J Epidemiol. 2017 Mar 27;46(6):2044–55. doi: 10.1093/ije/dyx022 (PMC5837355; doi:10.1093/ije/dyx022)
Supplement: Supplementary Data [file supplementary_material_dyx022.docx]

**SUPPLEMENTARY MATERIAL**

**SUPPLEMENTARY METHODS**

*1982 Pelotas Birth Cohort – Conventional association analysis*

*Body composition and anthropometric measures*

Abdominal fat depots were measured using the ultrasound machine Toshiba Xario (Toshiba Medical Systems Corp., Tokyo, Japan)^1-3^. Briefly, visceral fat thickness was estimated by the distance between the peritoneum and the lumbar spine at the intersection between the xyphoid line and the waist circumference. Subcutaneous abdominal fat thickness was estimated at the same probe site by the distance between the posterior line of dermis and the outer bowel wall. Intra-observer error was 4.1% for visceral and 3.4% for subcutaneous abdominal fat. Inter-observer technical error of measurement was 3.1% for both visceral fat and subcutaneous abdominal fat. Women that were pregnant or three months postpartum were excluded.

Gluteofemoral fat was assessed by Dual-energy X-ray Absorptiometry (DXA) (Lunar Prodigy Advance—GE, Germany). Participants with osteoarticular disabilities, confirmed or suspected pregnancy, non-removable metallic objects, wheelchair users, extremely obese (weight > 120 kg) or extremely tall (height > 192 cm) individuals were excluded. Weight was measured to the nearest 0.1 kg on a calibrated electronic scale (TANITA model BC‐418 MA; Tanita, Tokyo, Japan).

Standing height was assessed to the nearest 0.1 cm using a full‐length wall‐mounted stadiometer (SECA 240; Seca, Birmingham, United Kingdom).

*Genomic ancestry*

Genomic ancestry was estimated using 370,539 ancestry informative markers shared by samples from the HapMap Project ^4^, the Human Genome Diversity Project (HGDP) ^5^ and the Epigen-Brazil study population ^6^. The following HapMap samples were used: 266 Africans (176 Yoruba in Ibadan, Nigeria [YRI] and 90 Luhya in Webuye, Kenya [LWK]), 262 Europeans (174 Utah residents with Northern and Western European ancestry [CEU] and 88 from Toscans from Italy [TSI]), 170 admixed individuals (77 Mexicans from Los Angeles, California [MEX] and 83 Afro-African from Southwest USA [ASW]), and 93 Native Americans from the HGDP (25 Pima, 22 Karitiana, 25 Maya and 21 Surui). The software ADMIXTURE ^7^ was used to estimate the contribution from European, African and Native American ancestry for each cohort participant. SNPs were genotyped using Illumina Omni 2.5M-8v1 array (San Diego, California). Further details can be found in Lima-Costa et al. ^6^.

*GIANT and ADIPOGen consortia – Mendelian randomization analysis*

*Proportion of phenotypic variance explained by genetic instruments*

In order to estimate the strength of our genetic instruments, we estimated the phenotypic variance explained by a given SNP (R^2^) for each exposure of interest (waist circumference, hip circumference, and adiponectin concentration). We used ADIPOGen and GIANT summary data to approximate R^2^ for a given SNP based on the effect estimate for its association with the trait of interest (beta or $\hat{\beta}$), respective standard error ($se(\hat{\beta})$), minor allele frequency (MAF), and sample size (N). The following formula was used as previously described by Shim et al., 2015^8^:

$$R^{2} \cong\frac{2\hat{\beta}^{2}MAF(1-MAF)}{2\hat{\beta}^{2}MAF\left( 1-MAF \right)+(se({\hat{\beta}))}^{2}2NMAF\left( 1-MAF \right)}$$

The phenotypic variance explained by the composite genetic instrument (combining all SNPs) was estimated by the sum of SNP-specific R^2^.

*Power calculations*

We have estimated power for our Mendelian randomization analyses using the online calculator tool (<http://cnsgenomics.com/shiny/mRnd/>) and assuming a range of effect sizes for the potential underlying causal association between exposure and outcome. Details on the parameters used and the resulting estimated power are provided below.

| Exposure | Outcome | Sample size^1^ | Type-I error rate | Effect estimate^2^ | Instrument strength (R^2^)^3^ | Power |
| --- | --- | --- | --- | --- | --- | --- |
| WC | Adiponectin | 29,347 | 0.05 | 0.05 | 0.012 | 16% |
| WC | Adiponectin | 29,347 | 0.05 | 0.10 | 0.012 | 47% |
| WC | Adiponectin | 29,347 | 0.05 | 0.20 | 0.012 | 97% |
| HipC | Adiponectin | 29,347 | 0.05 | 0.05 | 0.02 | 23% |
| HipC | Adiponectin | 29,347 | 0.05 | 0.10 | 0.02 | 68% |
| HipC | Adiponectin | 29,347 | 0.05 | 0.20 | 0.02 | 100% |
| Adiponectin | WC or HipC | 210,088 | 0.05 | 0.05 | 0.04 | 100% |
| Adiponectin | WC or HipC | 210,088 | 0.05 | 0.10 | 0.04 | 100% |
| Adiponectin | WC or HipC | 210,088 | 0.05 | 0.20 | 0.04 | 100% |

^1^ Approximate sample size used for estimating SNP-outcome association

^2^ Considering the true underlying causal association is unknown, a range of values was used.

^3^ Instrument strength relates to the proportion of variance in the exposure explained by the instrument (R^2^). This was calculated by the sum of R^2^ from each SNP in the instrument (56 SNPs for waist circumference, 75 SNPs for hip circumference and 4 SNPs for adiponectin concentration). SNPs were in linkage equilibrium. The formula used to estimate R^2^ for each SNP is detailed in “*Proportion of phenotypic variance explained by genetic instruments”* section.

*Inverse-variance weighted (IVW) method*

For the unadjusted Mendelian randomization model, the inverse-variance weighted (IVW) method was used to derive the beta coefficient (mean difference in standardized log adiponectin per standard unit increase in waist or hip circumference) and its standard error by using the following formulas:

$$\hat{\beta}\mathrm{IVW} = \frac{\sum_{k=1}^{K} X_{k}Y_{k}\sigma_{yk}^{-2}}{\sum_{k=1}^{K} X_{k}^{2}\sigma_{yk}^{-2}} {SE}_{\hat{\beta}\mathrm{IVW}}= \sqrt{\frac{1}{\sum_{k=1}^{K} X_{k}^{2}\sigma_{yk}^{-2}}}$$

Where X_k_ is the mean difference in standardized waist or hip circumference per additional effect allele of SNP k and Y_k_ is the mean difference in standardized log adiponectin per additional effect allele of SNP k with standard error σ_Yk_.

For the adjusted Mendelian randomization model, we fitted a model having betas for SNP-adiponectin levels association as the dependent variable, betas for SNP-waist circumference and SNP-hip circumference as independent variables and inverse variance weights (with no intercept) to estimate the independent association of genetically increased waist or hip circumference with blood adiponectin levels. This method is equivalent to the unadjusted IVW method when there is only one independent variable ^9^.

In the original ADIPOGen summary dataset, betas for the association of SNPs with adiponectin concentration are provided as changes in log units of adiponectin per SNP allele. In order to have the same scale between Mendelian randomization and conventional association analysis, betas (and standard errors) from ADIPOGen dataset had to be harmonised prior to analysis. As only summary data was available, conversion of log adiponectin to equivalent standardized log adiponectin was made using individual level data from 1982 Pelotas Birth Cohort with similar adiponectin distribution (adiponectin levels in ADIPOGen consortium: mean = 9.8 μg/ml (SD = 5.6); adiponectin levels in 1982 Pelotas Birth Cohort: mean = 9.3 μg/ml (SD = 5.7)).

*MR-Egger regression method*

The Egger regression has been used for almost two decades to detect small study bias in meta-analyses of randomized clinical trials ^10^. In this method, the ratio of the effect estimate by its standard error is regressed against the estimate’s precision (the inverse of the standard error). Bowden et al. ^11^ recently proposed an adaptation of the Egger regression to test for bias from pleiotropy in Mendelian randomisation studies.

While the IVW estimate is equivalent to the slope of the best fitting line through the observations that pass through the origin, the MR-Egger estimate would be the of the best fitting line through the observations in a model that allows the intercept to vary. In this method, the intercept will reflect the average pleiotropic effect across genetic variants (e.g. mean difference in log adiponectin levels when difference in waist or hip circumference per allele is zero) and the slope coefficient will provide an estimate of the causal effect provided that the InSIDE (Instrument Strength Independent of Direct Effect) assumption holds, which requires that there is no correlation between SNP-exposure association and direct effects of SNP on outcome. The MR-Egger estimate may be underpowered, as it relies on variants having different strengths of association with the risk factor. Bootstrapping (10,000 iterations) was used to derive corrected 95% confidence intervals for MR-Egger intercept and slope using the percentile method ^11^.

*Penalized weighted median estimator*

Median-based methods give consistent estimates even when up to half the genetic variants are invalid instrumental variables. The weighted median estimate is defined as the median of an empirical distribution in which each instrumental variable estimate appears with probability proportional to the inverse of its variance^12^. The weighted median estimate is consistent under the assumption that genetic variants representing over 50% of the weight in the analysis are valid instruments. The contribution of heterogeneous variants to the weighted median estimate was downweighted (penalized) by multiplying the inverse-variance weight by the p-value of a chi-squared distribution (1 degree of freedom) corresponding to the *Q* statistics of each SNP when p-value < 0.05 ^13^. Bootstrapping (1,000 iterations) was carried out and the bootstrap standard error (the standard deviation of the bootstrap estimates) and a normal approximation (estimate ± 1.96*standard error) were used to derive 95% confidence interval ^13^.

**SUPPLEMENTARY TABLES AND FIGURES**

**Supplementary table 1.** Core instrumental variable assumptions and strategies used to address them

| *Assumption* | *Graphical examples of assumption violation^*^* | *Consequences of potential violation* | *Validation of assumption in the current analysis* |
| --- | --- | --- | --- |
| 1. **IV should be (strongly) associated with the exposure** | 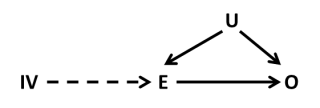 | A weak association between the IV and E can reduce precision and introduce weak instrument bias, which tends to bias the causal estimate towards the OLS estimate in one-sample MR | - Only genetic variants strongly associated with the exposure were selected  - In two-sample MR studies with non-overlapping datasets, any bias from weak instruments would be in the direction of the null and, thus, should not result in false positive findings |
| 1. **IV should only affect the outcome through the exposure** | 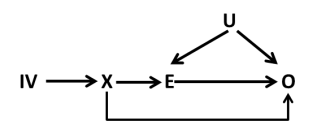  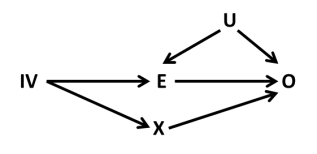  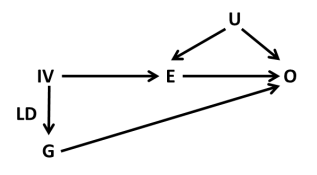 | Bias in MR estimate can result from horizontal pleiotropy (e.g. genetic variant itself or a correlated variant is associated with multiple pathways independent of the exposure) the direction and magnitude of this bias will depend up the direction and magnitude of the association path from IV to O that is not via E | - We extensively investigated heterogeneity and asymmetry in IVW estimates  - We compared results from the conventional Mendelian randomization analysis to other Mendelian randomization estimators (Penalized weighted median estimator MR-Egger method) based on a less stringent set of assumptions to assess the validity of our findings |
| 1. **IV should be independent of exposure-outcome confounders and IV-outcome exposures** | 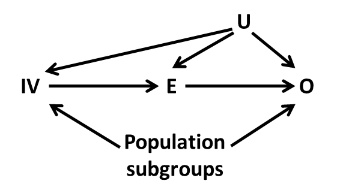 | In cases of population stratification, there could be an spurious association between IV and phenotypes | - To reduce the possibility of bias due to population stratification, the analyses were restricted to European-ancestry individuals  - All consortia accounted for population structure by adjusting for genomic control inflation factor |

IV: instrumental variable; E: exposure; O: outcome; U: unknown confounder; X: other phenotype: G: other genetic variant in LD; LD: linkage disequilibrium. A dashed arrow was used to indicate weak association between IV and E. Adapted from Vanderweele.^14^

**Supplementary Table 2.** Characteristics of data sources used in the Mendelian randomization analyses

| Consortium | ADIPOGen | GIANT |
| --- | --- | --- |
| Use | SNP-log adiponectin* | SNP-BMI-adjusted WC and SNP-BMI-adjusted HipC |
| Studies | 16 cohort studies with GWAS data | 101 studies of multiple designs with GWAS or Metabochip data |
| Study population | 29,347 individuals of European ancestry | ≈ 210,088 individuals of European ancestry |
| Study-specific mean age (in years) - median [range] | 52 [10, 75] | 58 [19, 76] |
| Study-specific mean adiponectin levels (µg/mL) - median [range] | 9.8 [4.9, 15.8] | N/A |
| Study-specific median WC (in cm) - median [range] | N/A | 101 [75, 116] |
| Study-specific median HipC (in cm) - median [range] | N/A | 96 [63, 119] |
| Imputation | IMPUTE, MACH, BIMBAM or Beagle (reference: Phase II CEU HapMap population) | IMPUTE, MACH or Beagle (reference: Phase II CEU HapMap population) |
| Quality control criteria† | Call rate > 0·95; MAF > 0·01; p _HWE_ > 10^-6^; and quality measures for imputed SNPs (r^2^ ≥ 0·3, or proper info ≥ 0·4) | Sample cal rate > 0·85-0·98; SNP call rate > 0·90-0·99; MAF > 0·00-0·01; p _HWE_ > 10^-3^-10^-7^; and quality measures for imputed SNPs (r^2^ ≥ 0·3, proper info ≥ 0·4, or no filtering) |
| Model | additive | additive |
| Adjustments | Age, sex, BMI, principal components of genomic ancestry, study site (where appropriate), family structure (one family-based study) and genomic control inflation factor (λ) | Age, age2, BMI and study specific variables (e.g. principal components of genomic ancestry), and genomic control inflation factor (λ) |
| Data download | <https://www.mcgill.ca/genepi/adipogen-consortium> | http://www.broadinstitute.org/collaboration/giant/index.php/GIANT_consortium_data_files |

* Blood adiponectin concentration was assessed using ELISA or RIA methods. † Quality control criteria may have varied across studies within each consortium. BMI: body mass index; CEU: Centre d’Etude du Polymorphisme Humain collected in Utah; GIANT: Genetic Investigation of ANthropometric Traits; GWAS: genome-wide association study; HipC: hip circumference; HWE: Hardy-Weinberg equilibrium; MAF: minor allele frequency; SNP: single nucleotide polymorphism; WC: waist circumference; N/A: not available. Information on study-specific age, adiponectin levels, WC, and HipC distribution were extracted from the supplementary material of the original publications of GIANT^15^ and ADIPOGen^16^. Medians were calculated based only on studies for which information was available in the original publications (all the 16 cohorts from ADIPOGen and 91 out of 101 studies from GIANT)

**Supplementary Table 3.** SNPs used as instrumental variables for waist circumference in Mendelian randomization analysis

| rs ID | Chr | EA | NEA | EAF | R^2^ | Beta | SE | P-value | N |
| --- | --- | --- | --- | --- | --- | --- | --- | --- | --- |
| rs9435732 | 1 | C | T | 0.825 | 0.0003 | 0.031 | 0.004 | 4E-16 | 228579 |
| rs7536458 | 1 | T | G | 0.65 | 0.0003 | 0.030 | 0.004 | 1E-15 | 228790 |
| rs12064744 | 1 | T | C | 0.3417 | 0.0002 | 0.026 | 0.004 | 2E-14 | 231298 |
| rs984222 | 1 | G | C | 0.575 | 0.0005 | 0.036 | 0.004 | 2E-25 | 231215 |
| rs11205277 | 1 | G | A | 0.3898 | 0.0003 | 0.027 | 0.004 | 1E-13 | 215898 |
| rs2274432 | 1 | A | G | 0.3729 | 0.0002 | 0.025 | 0.004 | 2E-12 | 227843 |
| rs12991495 | 2 | T | C | 0.675 | 0.0002 | 0.028 | 0.004 | 6E-14 | 229964 |
| rs6715793 | 2 | T | C | 0.45 | 0.0001 | 0.019 | 0.003 | 1E-08 | 231071 |
| rs2052670 | 2 | G | A | 0.4083 | 0.0001 | 0.020 | 0.004 | 2E-08 | 231210 |
| rs2124969 | 2 | C | T | 0.4083 | 0.0001 | 0.020 | 0.003 | 7E-09 | 231284 |
| rs13083798 | 3 | A | G | 0.5417 | 0.0002 | 0.020 | 0.003 | 3E-09 | 230391 |
| rs9864077 | 3 | T | C | 0.7583 | 0.0002 | 0.022 | 0.004 | 1E-09 | 219478 |
| rs6772896 | 3 | T | C | 0.6417 | 0.0002 | 0.024 | 0.004 | 2E-11 | 231246 |
| rs7621331 | 3 | A | G | 0.6917 | 0.0001 | 0.021 | 0.004 | 9E-09 | 231264 |
| rs1344674 | 3 | G | A | 0.4833 | 0.0002 | 0.024 | 0.003 | 4E-13 | 231241 |
| rs17451107 | 3 | T | C | 0.625 | 0.0002 | 0.026 | 0.004 | 1E-13 | 227636 |
| rs12493901 | 3 | G | A | 0.5417 | 0.0002 | 0.021 | 0.003 | 8E-10 | 230668 |
| rs710841 | 4 | T | C | 0.2417 | 0.0003 | 0.029 | 0.004 | 9E-14 | 230174 |
| rs17541471 | 5 | C | T | 0.2333 | 0.0001 | 0.023 | 0.004 | 4E-08 | 230478 |
| rs12656497 | 5 | T | C | 0.4833 | 0.0002 | 0.022 | 0.003 | 2E-10 | 231223 |
| rs459193 | 5 | A | G | 0.2167 | 0.0002 | 0.025 | 0.004 | 8E-11 | 231220 |
| rs10041657 | 5 | A | G | 0.2167 | 0.0002 | 0.025 | 0.004 | 3E-10 | 230824 |
| rs272869 | 5 | G | A | 0.6583 | 0.0002 | 0.021 | 0.003 | 7E-10 | 229935 |
| rs4868125 | 5 | G | C | 0.6417 | 0.0002 | 0.021 | 0.004 | 3E-09 | 225860 |
| rs10516107 | 5 | A | G | 0.2917 | 0.0002 | 0.023 | 0.004 | 8E-11 | 231310 |
| rs6556301 | 5 | T | G | 0.375 | 0.0003 | 0.028 | 0.004 | 2E-12 | 191245 |
| rs1776897 | 6 | G | T | 0.075 | 0.0004 | 0.061 | 0.007 | 6E-20 | 197374 |
| rs998584 | 6 | A | C | 0.475 | 0.0003 | 0.029 | 0.004 | 6E-15 | 210814 |
| rs395962 | 6 | T | G | 0.3667 | 0.0003 | 0.029 | 0.004 | 1E-15 | 231306 |
| rs2745359 | 6 | C | T | 0.069 | 0.0002 | 0.052 | 0.009 | 2E-09 | 178085 |
| rs2745353 | 6 | T | C | 0.55 | 0.0003 | 0.029 | 0.003 | 8E-19 | 231143 |
| rs6570507 | 6 | G | A | 0.75 | 0.0002 | 0.024 | 0.004 | 6E-11 | 228993 |
| rs798489 | 7 | C | T | 0.725 | 0.0002 | 0.025 | 0.004 | 1E-11 | 230932 |
| rs2214442 | 7 | G | A | 0.4417 | 0.0002 | 0.026 | 0.005 | 4E-09 | 152053 |
| rs4141278 | 7 | C | T | 0.1833 | 0.0003 | 0.034 | 0.004 | 3E-15 | 231233 |
| rs7801581 | 7 | T | C | 0.2583 | 0.0002 | 0.027 | 0.004 | 8E-11 | 216463 |
| rs849140 | 7 | T | C | 0.4 | 0.0003 | 0.029 | 0.003 | 5E-17 | 228910 |
| rs12679556 | 8 | G | T | 0.2083 | 0.0002 | 0.026 | 0.004 | 1E-11 | 225056 |
| rs7854560 | 9 | T | C | 0.2667 | 0.0002 | 0.026 | 0.004 | 5E-12 | 229674 |
| rs10748826 | 10 | T | C | 0.5776 | 0.0002 | 0.023 | 0.004 | 3E-10 | 195019 |
| rs2071449 | 12 | A | C | 0.325 | 0.0003 | 0.032 | 0.004 | 3E-18 | 226567 |
| rs7970350 | 12 | C | T | 0.5083 | 0.0001 | 0.019 | 0.003 | 4E-08 | 229815 |
| rs12317176 | 12 | T | C | 0.6167 | 0.0001 | 0.020 | 0.004 | 6E-09 | 230924 |
| rs12372180 | 12 | A | G | 0.0667 | 0.0001 | 0.041 | 0.007 | 3E-08 | 219175 |
| rs7166081 | 15 | A | G | 0.8083 | 0.0002 | 0.024 | 0.004 | 2E-09 | 230255 |
| rs4886782 | 15 | G | A | 0.7333 | 0.0002 | 0.024 | 0.004 | 6E-12 | 228446 |
| rs4246302 | 15 | G | A | 0.3333 | 0.0002 | 0.022 | 0.004 | 6E-09 | 227205 |
| rs4567683 | 15 | A | G | 0.2833 | 0.0001 | 0.022 | 0.004 | 8E-09 | 228589 |
| rs16957304 | 16 | A | G | 0.95 | 0.0002 | 0.059 | 0.011 | 3E-08 | 151917 |
| rs3760318 | 17 | G | A | 0.6417 | 0.0002 | 0.021 | 0.004 | 9E-10 | 228998 |
| rs757608 | 17 | A | G | 0.3 | 0.0002 | 0.027 | 0.004 | 1E-13 | 229039 |
| rs4239436 | 18 | G | A | 0.7417 | 0.0004 | 0.040 | 0.004 | 1E-22 | 229607 |
| rs12608504 | 19 | A | G | 0.3417 | 0.0001 | 0.020 | 0.004 | 2E-08 | 228998 |
| rs3786897 | 19 | G | A | 0.4083 | 0.0001 | 0.020 | 0.004 | 9E-09 | 228567 |
| rs979012 | 20 | T | C | 0.3583 | 0.0004 | 0.033 | 0.004 | 5E-20 | 229815 |
| rs2179129 | 22 | A | G | 0.55 | 0.0001 | 0.019 | 0.003 | 3E-08 | 228844 |

Chr: chromosome; EA: effect allele (trait-increasing allele); NEA: non-effect allele; R^2^: proportion of phenotypic variance explained by SNP; Beta: increase in standardized waist circumference per EA; SE: standard error; N: sample size.

**Supplementary Table 4.** SNPs used as instrumental variables for hip circumference in Mendelian randomization analysis

| rs ID | Chr | EA | NEA | EAF | R^2^ | Beta | SE | P-value | N |
| --- | --- | --- | --- | --- | --- | --- | --- | --- | --- |
| rs6657613 | 1 | T | A | 0.53 | 0.0004 | 0.031 | 0.004 | 4E-18 | 210917 |
| rs12086130 | 1 | T | C | 0.10 | 0.0002 | 0.037 | 0.006 | 3E-09 | 206610 |
| rs3748656 | 1 | C | T | 0.80 | 0.0002 | 0.024 | 0.004 | 6E-09 | 210890 |
| rs11205303 | 1 | C | T | 0.36 | 0.0005 | 0.041 | 0.004 | 6E-25 | 196314 |
| rs17346473 | 1 | G | A | 0.22 | 0.0003 | 0.030 | 0.004 | 3E-14 | 210431 |
| rs12075079 | 1 | G | A | 0.16 | 0.0002 | 0.031 | 0.004 | 5E-13 | 211016 |
| rs2301453 | 1 | A | G | 0.54 | 0.0002 | 0.022 | 0.004 | 6E-10 | 210882 |
| rs1046934 | 1 | C | A | 0.38 | 0.0002 | 0.023 | 0.004 | 6E-10 | 210450 |
| rs2820443 | 1 | C | T | 0.30 | 0.0007 | 0.048 | 0.004 | 2E-35 | 211030 |
| rs6672530 | 1 | A | C | 0.77 | 0.0002 | 0.028 | 0.005 | 8E-10 | 208172 |
| rs1545552 | 2 | G | A | 0.71 | 0.0003 | 0.029 | 0.004 | 6E-13 | 208132 |
| rs10195252 | 2 | C | T | 0.44 | 0.0002 | 0.023 | 0.004 | 1E-10 | 210403 |
| rs4973517 | 2 | T | C | 0.75 | 0.0002 | 0.029 | 0.005 | 2E-10 | 175930 |
| rs11242 | 3 | T | C | 0.43 | 0.0003 | 0.027 | 0.004 | 6E-14 | 204637 |
| rs1388251 | 3 | A | G | 0.74 | 0.0002 | 0.023 | 0.004 | 2E-08 | 211029 |
| rs10804591 | 3 | C | A | 0.15 | 0.0004 | 0.038 | 0.004 | 7E-18 | 210953 |
| rs724016 | 3 | G | A | 0.48 | 0.0009 | 0.048 | 0.004 | 8E-43 | 211032 |
| rs4243400 | 3 | G | A | 0.50 | 0.0002 | 0.025 | 0.004 | 3E-12 | 210478 |
| rs2098771 | 3 | G | A | 0.33 | 0.0001 | 0.022 | 0.004 | 4E-08 | 196732 |
| rs6845078 | 4 | C | T | 0.84 | 0.0002 | 0.035 | 0.005 | 9E-12 | 207534 |
| rs9993613 | 4 | T | G | 0.51 | 0.0003 | 0.027 | 0.005 | 7E-10 | 143494 |
| rs1662837 | 4 | C | T | 0.28 | 0.0003 | 0.028 | 0.004 | 1E-13 | 210825 |
| rs12648786 | 4 | A | G | 0.41 | 0.0003 | 0.032 | 0.004 | 2E-16 | 199289 |
| rs11736535 | 4 | G | A | 0.30 | 0.0003 | 0.029 | 0.005 | 1E-10 | 143695 |
| rs11730399 | 4 | A | C | 0.95 | 0.0003 | 0.060 | 0.008 | 5E-13 | 173372 |
| rs1173771 | 5 | A | G | 0.47 | 0.0002 | 0.026 | 0.004 | 6E-13 | 210986 |
| rs7703857 | 5 | T | C | 0.41 | 0.0003 | 0.028 | 0.005 | 5E-10 | 143721 |
| rs1294410 | 6 | T | C | 0.38 | 0.0003 | 0.029 | 0.004 | 2E-15 | 210861 |
| rs13216391 | 6 | G | A | 0.15 | 0.0002 | 0.028 | 0.005 | 3E-09 | 199240 |
| rs11754288 | 6 | A | G | 0.47 | 0.0002 | 0.021 | 0.004 | 3E-09 | 210954 |
| rs12210905 | 6 | A | G | 0.88 | 0.0002 | 0.033 | 0.006 | 1E-08 | 210929 |
| rs1759645 | 6 | C | T | 0.13 | 0.0002 | 0.029 | 0.005 | 9E-09 | 209671 |
| rs16894959 | 6 | C | T | 0.10 | 0.0002 | 0.036 | 0.005 | 3E-13 | 210242 |
| rs975496 | 6 | G | A | 0.84 | 0.0002 | 0.031 | 0.005 | 1E-09 | 199331 |
| rs6903448 | 6 | C | T | 0.84 | 0.0002 | 0.034 | 0.005 | 5E-12 | 211031 |
| rs12207675 | 6 | C | T | 0.13 | 0.0003 | 0.041 | 0.006 | 1E-13 | 211077 |
| rs7759938 | 6 | C | T | 0.36 | 0.0003 | 0.028 | 0.004 | 2E-13 | 211029 |
| rs1538170 | 6 | T | C | 0.38 | 0.0002 | 0.026 | 0.004 | 3E-12 | 201926 |
| rs9491696 | 6 | C | G | 0.47 | 0.0002 | 0.023 | 0.004 | 1E-10 | 210813 |
| rs9388766 | 6 | T | C | 0.33 | 0.0002 | 0.028 | 0.004 | 8E-13 | 211072 |
| rs6570509 | 6 | G | T | 0.74 | 0.0006 | 0.045 | 0.004 | 1E-29 | 197803 |
| rs798497 | 7 | A | G | 0.72 | 0.0004 | 0.035 | 0.004 | 4E-20 | 210942 |
| rs849141 | 7 | A | G | 0.29 | 0.0003 | 0.032 | 0.004 | 2E-16 | 211081 |
| rs42235 | 7 | T | C | 0.34 | 0.0004 | 0.036 | 0.004 | 8E-20 | 208455 |
| rs3731321 | 7 | T | C | 0.87 | 0.0002 | 0.029 | 0.005 | 5E-08 | 182525 |
| rs7008867 | 8 | A | G | 0.21 | 0.0002 | 0.024 | 0.004 | 6E-09 | 211066 |
| rs10958476 | 8 | C | T | 0.14 | 0.0002 | 0.028 | 0.005 | 9E-10 | 199716 |
| rs6984782 | 8 | T | C | 0.88 | 0.0002 | 0.033 | 0.005 | 2E-09 | 210592 |
| rs6470764 | 8 | C | T | 0.81 | 0.0004 | 0.039 | 0.005 | 8E-18 | 210864 |
| rs7007820 | 8 | A | G | 0.63 | 0.0001 | 0.020 | 0.004 | 1E-08 | 211016 |
| rs4448343 | 9 | G | A | 0.32 | 0.0002 | 0.024 | 0.004 | 3E-11 | 210984 |
| rs10123368 | 9 | C | T | 0.20 | 0.0002 | 0.026 | 0.004 | 5E-09 | 210933 |
| rs686320 | 11 | G | C | 0.91 | 0.0002 | 0.038 | 0.006 | 7E-12 | 199308 |
| rs1351394 | 12 | T | C | 0.48 | 0.0002 | 0.025 | 0.004 | 5E-13 | 210068 |
| rs10748128 | 12 | T | G | 0.36 | 0.0002 | 0.023 | 0.004 | 4E-09 | 197305 |
| rs7953508 | 12 | T | C | 0.25 | 0.0002 | 0.024 | 0.004 | 4E-09 | 210624 |
| rs12817549 | 12 | T | C | 0.57 | 0.0003 | 0.029 | 0.004 | 2E-16 | 210856 |
| rs1727294 | 12 | A | G | 0.20 | 0.0003 | 0.032 | 0.004 | 8E-14 | 208707 |
| rs3118906 | 13 | G | A | 0.76 | 0.0003 | 0.030 | 0.004 | 8E-15 | 211005 |
| rs558003 | 13 | A | G | 0.04 | 0.0003 | 0.049 | 0.006 | 4E-15 | 199267 |
| rs10140922 | 14 | G | T | 0.63 | 0.0003 | 0.030 | 0.005 | 5E-11 | 143568 |
| rs1254263 | 14 | C | T | 0.28 | 0.0003 | 0.029 | 0.005 | 9E-10 | 143808 |
| rs17193922 | 16 | G | C | 0.38 | 0.0002 | 0.024 | 0.004 | 1E-09 | 198589 |
| rs9890032 | 17 | C | G | 0.63 | 0.0002 | 0.026 | 0.004 | 2E-12 | 207385 |
| rs561341 | 17 | G | T | 0.17 | 0.0002 | 0.031 | 0.005 | 3E-10 | 211106 |
| rs7223966 | 17 | A | G | 0.32 | 0.0003 | 0.029 | 0.004 | 2E-13 | 211080 |
| rs1120297 | 17 | T | C | 0.48 | 0.0002 | 0.021 | 0.004 | 2E-09 | 210991 |
| rs4369779 | 18 | C | T | 0.74 | 0.0003 | 0.035 | 0.004 | 3E-15 | 210787 |
| rs181553 | 18 | A | G | 0.68 | 0.0003 | 0.029 | 0.004 | 9E-15 | 210832 |
| rs12980348 | 19 | G | T | 0.38 | 0.0003 | 0.029 | 0.004 | 9E-16 | 210456 |
| rs169797 | 20 | A | G | 0.75 | 0.0002 | 0.024 | 0.004 | 1E-09 | 204594 |
| rs6088619 | 20 | G | A | 0.13 | 0.0003 | 0.039 | 0.005 | 9E-13 | 199166 |
| rs143384 | 20 | G | A | 0.40 | 0.0006 | 0.044 | 0.004 | 1E-31 | 209682 |
| rs6060717 | 20 | C | T | 0.16 | 0.0002 | 0.032 | 0.005 | 6E-12 | 211073 |
| rs6141600 | 20 | C | T | 0.28 | 0.0003 | 0.035 | 0.005 | 3E-11 | 142740 |

Chr: chromosome; EA: effect allele (trait-increasing allele); NEA: non-effect allele; R^2^: proportion of phenotypic variance explained by SNP; Beta: increase in standardized hip circumference per EA; SE: standard error; N: sample size.

**Supplementary Table 4 (continued)**

**Supplementary Table 5.** SNPs used as instrumental variables for adiponectin concentration in Mendelian randomisation analysis and association with adiponectin concentration

| rs ID | Chr | EA | NEA | EAF | R^2^ | Beta | SE | P-value | N |
| --- | --- | --- | --- | --- | --- | --- | --- | --- | --- |
| rs6810075 | 3 | T | C | 0.63 | 0.0066 | 0.108 | 0.0078 | 4.E-41 | 29140 |
| rs16861209 | 3 | A | C | 0.08 | 0.0125 | 0.313 | 0.0163 | 3.E-77 | 29199 |
| rs17366568 | 3 | G | A | 0.91 | 0.0125 | 0.252 | 0.0142 | 3.E-66 | 24865 |
| rs3774261 | 3 | A | G | 0.40 | 0.0080 | 0.114 | 0.0075 | 1.E-49 | 29081 |

Chr: chromosome; EA: effect allele (trait-increasing allele); NEA: non-effect allele; R^2^: proportion of phenotypic variance explained by SNP; Beta: increase in standardized log adiponectin concentration per EA; SE: standard error; N: sample size.

**Supplementary Table 6**. Association of fat depots and adiponectin concentration with covariates according to sex

|  | Visceral fat | | | Deep subcutaneous abdominal fat | | | Superficial subcutaneous abdominal fat | | | Gluteofemoral fat | | | Adiponectin concentration | | |
| --- | --- | --- | --- | --- | --- | --- | --- | --- | --- | --- | --- | --- | --- | --- | --- |
|  | β | 95% CI | | β | 95% CI | | β | 95% CI | | β | 95% CI | | β | 95% CI | |
|  | *Males* | | | | | | | | | | | | | | |
| African ancestry (%) |  |  |  |  |  |  |  |  |  |  |  |  |  |  |  |
| 0.00-4.59 | Ref |  |  | Ref |  |  | Ref |  |  | Ref |  |  | Ref |  |  |
| 4.60-10.99 | 0.01 | -0.12 | 0.13 | -0.02 | -0.14 | 0.10 | -0.03 | -0.16 | 0.10 | -0.09 | -0.22 | 0.04 | 0.00 | -0.13 | 0.14 |
| 11.00-87.91 | -0.11 | -0.24 | 0.02 | -0.19 | -0.31 | -0.07 | -0.06 | -0.18 | 0.07 | -0.27 | -0.40 | -0.14 | -0.12 | -0.25 | 0.01 |
| Leisure-time physical activity | |  |  |  |  |  |  |  |  |  |  |  |  |  |  |
| Inactive | Ref |  |  | Ref |  |  | Ref |  |  | Ref |  |  | Ref |  |  |
| Insufficiently active | -0.02 | -0.15 | 0.11 | -0.02 | -0.15 | 0.11 | -0.08 | -0.22 | 0.05 | -0.08 | -0.22 | 0.06 | 0.04 | -0.10 | 0.18 |
| Active | -0.18 | -0.30 | -0.06 | -0.10 | -0.22 | 0.02 | -0.09 | -0.21 | 0.04 | -0.13 | -0.26 | 0.00 | -0.07 | -0.20 | 0.06 |
| Smoking |  |  |  |  |  |  |  |  |  |  |  |  |  |  |  |
| Never smoker | Ref |  |  | Ref |  |  | Ref |  |  | Ref |  |  | Ref |  |  |
| Ex-smoker | 0.13 | -0.01 | 0.27 | -0.10 | -0.24 | 0.03 | -0.17 | -0.31 | -0.03 | -0.05 | -0.20 | 0.10 | -0.04 | -0.19 | 0.11 |
| 1-9 cigarettes/day | -0.04 | -0.23 | 0.16 | -0.33 | -0.51 | -0.14 | -0.37 | -0.57 | -0.18 | -0.39 | -0.60 | -0.19 | 0.00 | -0.21 | 0.20 |
| ≥ 10 cigarettes/day | -0.03 | -0.17 | 0.11 | -0.39 | -0.52 | -0.26 | -0.46 | -0.59 | -0.32 | -0.40 | -0.55 | -0.26 | 0.04 | -0.11 | 0.19 |
| Alcohol drinking |  |  |  |  |  |  |  |  |  |  |  |  |  |  |  |
| < 1 dose/day | Ref |  |  | Ref |  |  | Ref |  |  | Ref |  |  | Ref |  |  |
| ≥ 1 dose/day | 0.18 | 0.08 | 0.29 | -0.03 | -0.14 | 0.07 | -0.09 | -0.20 | 0.02 | 0.01 | -0.11 | 0.12 | 0.00 | -0.11 | 0.12 |
|  |  | | | | | | | | | | | | | | |
|  | *Females* | | | | | | | | | | | | | | |
| African ancestry (%) |  |  |  |  |  |  |  |  |  |  |  |  |  |  |  |
| 0.00-4.59 | Ref |  |  | Ref |  |  | Ref |  |  | Ref |  |  | Ref |  |  |
| 4.60-10.99 | 0.06 | -0.07 | 0.18 | -0.06 | -0.18 | 0.07 | -0.02 | -0.15 | 0.10 | -0.13 | -0.26 | 0.00 | -0.13 | -0.26 | -0.01 |
| 11.00-87.91 | 0.21 | 0.09 | 0.33 | 0.02 | -0.10 | 0.15 | 0.10 | -0.02 | 0.23 | -0.03 | -0.16 | 0.10 | -0.27 | -0.39 | -0.14 |
| Leisure-time physical activity | |  |  |  |  |  |  |  |  |  |  |  |  |  |  |
| Inactive | Ref |  |  | Ref |  |  | Ref |  |  | Ref |  |  | Ref |  |  |
| Insufficiently active | -0.21 | -0.35 | -0.07 | 0.02 | -0.12 | 0.16 | 0.03 | -0.11 | 0.17 | -0.05 | -0.19 | 0.10 | 0.02 | -0.13 | 0.16 |
| Active | -0.30 | -0.42 | -0.17 | -0.08 | -0.21 | 0.05 | -0.10 | -0.23 | 0.02 | -0.07 | -0.20 | 0.06 | 0.09 | -0.04 | 0.22 |
| Smoking |  |  |  |  |  |  |  |  |  |  |  |  |  |  |  |
| Never smoker | Ref |  |  | Ref |  |  | Ref |  |  | Ref |  |  | Ref |  |  |
| Ex-smoker | 0.07 | -0.07 | 0.20 | -0.02 | -0.16 | 0.12 | -0.03 | -0.16 | 0.11 | -0.08 | -0.22 | 0.06 | -0.11 | -0.25 | 0.02 |
| 1-9 cigarettes/day | -0.04 | -0.22 | 0.14 | 0.00 | -0.18 | 0.19 | -0.16 | -0.34 | 0.02 | -0.28 | -0.47 | -0.09 | -0.14 | -0.33 | 0.04 |
| ≥ 10 cigarettes/day | 0.15 | 0.00 | 0.31 | -0.13 | -0.29 | 0.03 | -0.25 | -0.40 | -0.09 | -0.31 | -0.47 | -0.15 | -0.28 | -0.43 | -0.12 |
| Alcohol drinking |  |  |  |  |  |  |  |  |  |  |  |  |  |  |  |
| < 1 dose/day | Ref |  |  | Ref |  |  | Ref |  |  | Ref |  |  | Ref |  |  |
| ≥ 1 dose/day | -0.01 | -0.12 | 0.09 | 0.04 | -0.07 | 0.15 | -0.04 | -0.14 | 0.07 | 0.05 | -0.06 | 0.16 | 0.06 | -0.05 | 0.16 |

Fat depots and adiponectin concentration are expressed as standard deviation units.

**Supplementary Table 7**. Pearson’s correlation coefficients between different measures of adiposity

|  |  | BMI | Total fat | VAT | dSCAAT | sSCAAT | GFAT |
| --- | --- | --- | --- | --- | --- | --- | --- |
| MALE | BMI | 1.00 | 0.89 | 0.67 | 0.66 | 0.60 | 0.84 |
|  | Total fat | 0.89 | 1.00 | 0.62 | 0.75 | 0.71 | 0.97 |
|  | VAT | 0.67 | 0.62 | 1.00 | 0.35 | 0.30 | 0.53 |
|  | dSCAAT | 0.66 | 0.75 | 0.35 | 1.00 | 0.59 | 0.71 |
|  | sSCAAT | 0.60 | 0.71 | 0.30 | 0.59 | 1.00 | 0.69 |
|  | GFAT | 0.84 | 0.97 | 0.53 | 0.71 | 0.69 | 1.00 |
| FEMALE | BMI | 1.00 | 0.94 | 0.63 | 0.71 | 0.66 | 0.89 |
|  | Total fat | 0.94 | 1.00 | 0.55 | 0.73 | 0.68 | 0.97 |
|  | VAT | 0.63 | 0.55 | 1.00 | 0.34 | 0.31 | 0.48 |
|  | dSCAAT | 0.71 | 0.73 | 0.34 | 1.00 | 0.46 | 0.65 |
|  | sSCAAT | 0.66 | 0.68 | 0.31 | 0.46 | 1.00 | 0.61 |
|  | GFAT | 0.89 | 0.97 | 0.48 | 0.65 | 0.61 | 1.00 |

BMI: body mass index; GFAT: gluteofemoral adipose tissue; dSCAAT: deep subcutaneous adipose tissue; sSCAAT: superficial subcutaneous adipose tissue; VAT: visceral adipose tissue. Data from the 2012 follow-up of the 1982 Pelotas Birth Cohort.

**Supplementary Table 8**. P values for the association of study variables with missingness indicator

|  | P values | |
| --- | --- | --- |
| Variable | Males | Females |
| African ancestry | 0.13 | 0.64 |
| Leisure-time physical activity | 0.10 | 0.02 |
| Smoking | 0.47 | 0.48 |
| Alcohol drinking | 0.03 | 0.70 |
| Body mass index | **1*10^-10^** | 0.74 |
| Visceral fat | **2*10^-4^** | 0.74 |
| Deep subcutaneous abdominal fat | **3*10^-5^** | 0.72 |
| Superficial subcutaneous abdominal fat | **0.01** | 0.41 |
| Gluteofemoral fat | 0.16 | 0.34 |
| Adiponectin | 0.80 | 0.67 |
| Glucose | 0.12 | 0.83 |
| C reactive protein | 0.16 | 0.19 |

Data from the 2012 follow-up of the 1982 Pelotas Birth Cohort

**Supplementary Figure 1.** Metanalysis and heterogeneity analysis of Mendelian randomization estimates of each SNP for the association of waist circumference with blood adiponectin levels. Data from GIANT (n = up to 210,088 individuals) and ADIPOGen (n = 29,347 individuals) consortia.

**Supplementary Figure 2.** Metanalysis and heterogeneity analysis of Mendelian randomization estimates of each SNP for the association of hip circumference with blood adiponectin levels. Data from GIANT (n = up to 210,088 individuals) and ADIPOGen (n = 29,347 individuals) consortia.

**Supplementary Figure 3.** Funnel plot of instrument precision (standard error of IVW estimate) against IVW estimates for each genetic variant for Mendelian randomization analysis of the influence of waist (A) or hip (B) circumference on adiponectin levels. Each blue dot corresponds to estimates of one genetic variant. Full vertical line represents the overall IVW estimate and dashed lines represent pseudo 95% confidence limits. Red line indicates the presence of asymmetry. IVW: inverse-variance weighted method. Data from GIANT (n = up to 210,088 individuals) and ADIPOGen (n = 29,347 individuals) consortia.

References

1. Rolfe Ede L, Loos RJ, Druet C, et al. Association between birth weight and visceral fat in adults. *Am J Clin Nutr* 2010; **92**: 347-52.

2. Stolk RP, Wink O, Zelissen PM, Meijer R, van Gils AP, Grobbee DE. Validity and reproducibility of ultrasonography for the measurement of intra-abdominal adipose tissue. *Int J Obes Relat Metab Disord* 2001; **25**: 1346-51.

3. Araujo de Franca GV, Lucia Rolfe E, Horta BL, et al. Associations of birth weight, linear growth and relative weight gain throughout life with abdominal fat depots in adulthood: the 1982 Pelotas (Brazil) birth cohort study. *Int J Obes (Lond)* 2015.

4. International HapMap C, Altshuler DM, Gibbs RA, et al. Integrating common and rare genetic variation in diverse human populations. *Nature* 2010; **467**: 52-8.

5. Li JZ, Absher DM, Tang H, et al. Worldwide human relationships inferred from genome-wide patterns of variation. *Science* 2008; **319**: 1100-4.

6. Lima-Costa MF, Rodrigues LC, Barreto ML, et al. Genomic ancestry and ethnoracial self-classification based on 5,871 community-dwelling Brazilians (The Epigen Initiative). *Sci Rep* 2015; **5**: 9812.

7. Alexander DH, Novembre J, Lange K. Fast model-based estimation of ancestry in unrelated individuals. *Genome Res* 2009; **19**: 1655-64.

8. Shim H, Chasman DI, Smith JD, et al. A multivariate genome-wide association analysis of 10 LDL subfractions, and their response to statin treatment, in 1868 Caucasians. *PLoS One* 2015; **10**: e0120758.

9. Burgess S, Dudbridge F, Thompson SG. Re: "Multivariable Mendelian randomization: the use of pleiotropic genetic variants to estimate causal effects". *Am J Epidemiol* 2015; **181**: 290-1.

10. Egger M, Davey Smith G, Schneider M, Minder C. Bias in meta-analysis detected by a simple, graphical test. *BMJ* 1997; **315**: 629-34.

11. Bowden J, Davey Smith G, Burgess S. Mendelian randomization with invalid instruments: effect estimation and bias detection through Egger regression. *Int J Epidemiol* 2015; **44**: 512-25.

12. Bowden J, Davey Smith G, Haycock PC, Burgess S. Consistent Estimation in Mendelian Randomization with Some Invalid Instruments Using a Weighted Median Estimator. *Genet Epidemiol* 2016; **40**: 304-14.

13. Bowden J, Smith GD, Haycock PC, Burgess S. Consistent estimation in Mendelian randomization with some invalid instruments using a weighted median estimator. *Genetic Epidemiology (in press)* 2016.

14. VanderWeele TJ, Tchetgen Tchetgen EJ, Cornelis M, Kraft P. Methodological challenges in mendelian randomization. *Epidemiology* 2014; **25**: 427-35.

15. Shungin D, Winkler TW, Croteau-Chonka DC, et al. New genetic loci link adipose and insulin biology to body fat distribution. *Nature* 2015; **518**: 187-96.

16. Dastani Z, Hivert MF, Timpson N, et al. Novel loci for adiponectin levels and their influence on type 2 diabetes and metabolic traits: a multi-ethnic meta-analysis of 45,891 individuals. *PLoS Genet* 2012; **8**: e1002607.
